# The Effects of Pterostilbene on Neutrophil Activity in Experimental Model of Arthritis

**DOI:** 10.1155/2013/106041

**Published:** 2013-09-30

**Authors:** Tomas Perecko, Katarina Drabikova, Antonin Lojek, Milan Ciz, Silvester Ponist, Katarina Bauerova, Radomir Nosal, Juraj Harmatha, Viera Jancinova

**Affiliations:** ^1^Institute of Experimental Pharmacology and Toxicology, Slovak Academy of Sciences, Dubravska Cesta 9, 841 04 Bratislava, Slovakia; ^2^Institute of Biophysics, Academy of Sciences of the Czech Republic, v. v. i., Kralovopolska 135, 612 65 Brno, Czech Republic; ^3^Institute of Organic Chemistry and Biochemistry, Academy of Sciences of the Czech Republic, v.v.i., Flemingovo Namesti 2, 166 10 Prague, Czech Republic

## Abstract

It has been demonstrated that pterostilbene inhibits reactive oxygen species production in neutrophils *in vitro*. However, little is known about its effects on neutrophils during inflammation *in vivo*. In this study, the effect of pterostilbene on neutrophil activity was investigated in experimental arthritis model. Lewis rats were injected by a single intradermal injection of heat-killed *Mycobacterium butyricum* in Freund's adjuvant to develop arthritis. Another group of arthritic animals received pterostilbene 30 mg/kg, daily, p.o. The number and activity of neutrophils in blood were measured on a weekly basis during the whole experiment. Moreover, the total radical trapping potential in plasma was measured at the end of the experiment. In the pterostilbene treated arthritic group, the treatment significantly lowered the number of neutrophils in blood on days 14 and 21 without significant downregulation of neutrophil oxidative burst. Pterostilbene nonsignificantly increased total radical trapping potential in arthritic animals. These results indicate that the promising effects of pterostilbene on reactive oxygen species operate by different mechanisms *in vitro* and in the animal model of inflammation. In conclusion, the positive effects of pterostilbene in the model of arthritis may be attributed to regulation of neutrophil number.

## 1. Introduction

Neutrophils are a crucial part of the innate immune system. Once activated, they adhere to and migrate through the endothelium to get to the inflamed tissue. Neutrophils contain NADPH-oxidase which generates superoxide anion [1]. In response to infection or foreign particles, neutrophils produce reactive oxygen species (ROS) to destroy the invading pathogens, which is known as oxidative burst of neutrophils [2]. The dark side of neutrophil activity is its contribution to tissue damage. This is due to overproduction of ROS seen in many inflammatory diseases, for example, rheumatoid arthritis [3–5].

**Figure 1:**
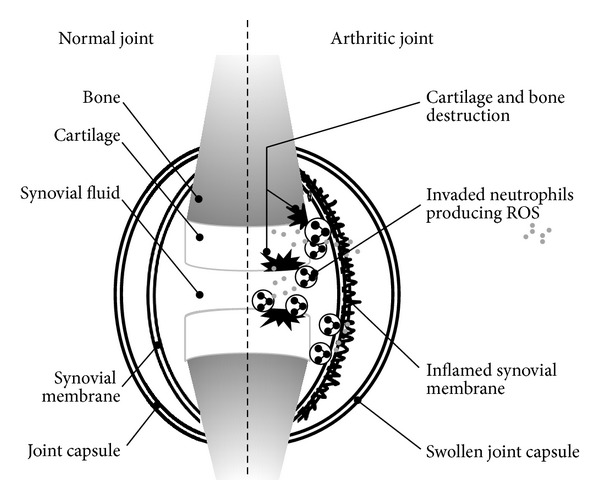
The role of neutrophils in pathogenesis of arthritis.

Rheumatoid arthritis is a chronic autoimmune inflammatory disease characterised by bone erosion and cartilage damage with synovial hyperplasia and pain [6]. Neutrophils are present in the synovial fluid and on the pannus-cartilage interface in arthritis [7, 8]. In addition, isolated neutrophils from arthritic patients showed preactivation (priming) which may be due to the presence of different cytokines in the synovial fluid [3]. Thus by producing ROS, activated neutrophils could contribute to joint destruction (Figure 1). This highlights the importance of searching for therapeutic agents capable of controlling the oxidative burst of neutrophils in neutrophil-dominant inflammatory diseases. Recently, significant research interest has been focused on resolution as a means to treat inflammatory diseases [9]. Resolution was thought to be a passive process caused by reduction of proinflammatory chemokines. Now, however, it is believed to be an active process with significant impact on neutrophil functionality [10, 11]. During resolution, in particular, the activity of neutrophils is downregulated [12]. To study the influence of neutrophils on pathogenesis of rheumatoid arthritis and to investigate the effects of new compounds on this process, different animal models of arthritis are used. One of them is the model of adjuvant arthritis induced by heat-inactivated *Mycobacterium butyricum* in Freund's adjuvant [13]. Mohr et al. showed that under normal conditions, there were no neutrophils in the articular capsule of nonarthritic animals, while the inflamed synovial and capsular tissue of adjuvant injected animals is heavily infiltrated with neutrophils [7]. We used this *in vivo* model of rat adjuvant arthritis to study whether pterostilbene—a stilbene type polyphenol—was capable of downregulating the activity of neutrophils.

**Figure 2:**
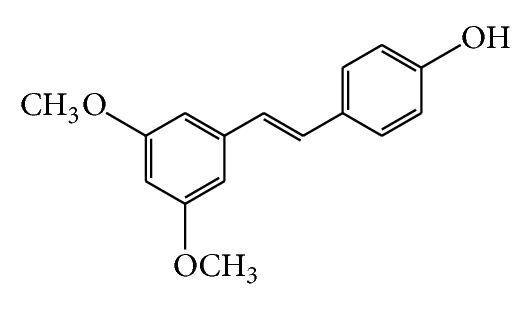
Structure of pterostilbene.

Pterostilbene (*trans*-3,5-dimethoxy-4′-hydroxystilbene, Figure 2) is a dimethyl derivative of resveratrol (*trans*-3,5,4′-trihydroxystilbene). The natural sources of pterostilbene are various herbal drugs, for example, leaves and grapes of *Vitis vinifera*, *Vaccinium *spp., and *Pterocarpus marsupium* [14–16]. Pterostilbene was found to have anti-inflammatory, antidiabetic, antifungal, and anticancerous effects [17, 18]. It was reported to inhibit lipopolysaccharide induced expression of COX-2 [19]. In our previous experiments, pterostilbene was the most effective resveratrol derivative tested in inhibiting ROS production by human neutrophils *in vitro*. Moreover, pterostilbene decreased the production of extracellular ROS, similarly as did resveratrol. Yet on intracellular ROS production, the effect of pterostilbene was lower in comparison with resveratrol [20]. Extracellular ROS are responsible for the tissue destruction, whereas intracellular ROS are involved in killing pathogens and in intracellular signalling [21].

In this study we investigated the effects of pterostilbene on the oxidative burst of neutrophils in arthritic rats. According to our knowledge, no papers have been published on the effects of pterostilbene on neutrophil activity in an *in vivo* model of chronic inflammation.

## 2. Materials and Method

Pterostilbene (precisely specified as *trans*-pterostilbene in this paper) was synthesised at the Institute of Chemical Technology, Prague, Czech Republic, and structurally characterised as (E)-4′-hydroxy-3,5-dimethoxystilbene in the Institute of Organic Chemistry and Biochemistry, Prague, Czech Republic [22]. TLC, RP-HPLC, and ^1^H-NMR spectroscopy were used for standardisation before and during the experiments.

2,2-Azo-bis-2-amidinopropane dihydrochloride (AAPH), horseradish peroxidase (HRP), luminol, phorbol myristate acetate (PMA), trichloroacetic acid, and Trolox were purchased from Sigma, Germany. *Mycobacterium butyricum *obtained from Difco Laboratories (Detroit, MI, USA) was suspended in Freund's adjuvant. All other products are available commercially or their origin is mentioned in the text.

### 2.1. Model of Adjuvant Arthritis in Rats

The study was performed in compliance with Principles of Laboratory Animal Care and was approved by the institutional Ethics Committee and by the State Veterinary and Food Administration of the Slovak Republic (Ro-1668/09-221). Animals were kept in an air-conditioned room with 12 hours day/night mode and drinking water *ad libitum*.

Male Lewis rats (Dobra Voda, Slovaki) were injected by a single intradermal injection of heat-killed *Mycobacterium butyricum* in Freund's adjuvant to develop arthritis [23]. Healthy control group and arthritic control group were treated with the solvent agent-sunflower oil. The doses of pterostilbene used in *in vivo* studies with rats range from 10 to 40 mg/kg [24–26]. In our experiment, pterostilbene 30 mg/kg, daily, p.o. in sunflower oil, was applied over a period of 21 days after arthritis induction. There were 10 animals in each group. On days 0, 7, 14, and 21, whole blood (10 *µ*L) from the tip of the tail was taken to citrated pipette tip and immediately diluted in Tyrode solution. The procedure was performed under local anaesthesia. The blood was subjected to further analysis of chemiluminescence and neutrophil numbers. After the experiment, the animals were sacrificed by overdosing with ketamine/xylazine anaesthesia. The blood was taken by cardiac puncture and plasma was obtained by 2000 g/15 min centrifugation for further analysis of total peroxyl radical trapping capacity (TRAP).

### 2.2. Evaluation of Neutrophil Number and ROS Production in Rats

The number of neutrophils in rat blood was calculated with a haemocytometer (Beckman Coulter). Spontaneous and PMA-stimulated oxidative burst of neutrophils in rat blood were determined by using chemiluminescence. Briefly chemiluminescence of neutrophils in blood was measured in a 96-well microplate luminometer (LM-01T Immunotech) at 37°C. Aliquots of Tyrode buffer and luminol (250 *µ*mol/L) were added. To ensure sufficient concentration of extracellular peroxidase in stimulated cells, we added horseradish peroxidase (HRP) to the final concentration 8 U/mL. Finally, 50-times diluted blood was added and the reaction was started by adding phorbol myristate acetate (PMA) to the final concentrations of 0.005–0.5 *µ*mol/L [27]. The chemiluminescence of the samples was recorded for 1 hour and area under the curve was examined. Normalisation of the oxidative burst of neutrophils in rat blood to the number of neutrophils in rat blood was calculated by using

(1) neutrophil  activity  =CL  of  samplenumber  of  neutrophils  in  the  sample  

(CL: chemiluminescence).

### 2.3. Total Peroxyl Radical Trapping Capacity (TRAP) of Rat Plasma

By using thermal decomposition of 2,2-azo-bis-2-amidinopropane dihydrochloride (AAPH), peroxyl radicals were monitored by luminol-enhanced chemiluminescence [28]. The reaction mixture consisted of 480 *µ*L of PBS and 50 *µ*L of 10 mmol/L luminol. Then 20 *µ*L of plasma samples or Trolox was added directly into the cuvette and the samples were preincubated for 10 min/37°C. Finally, 50 *µ*L of AAPH was added directly into the cuvette. Time needed for a 50% recovery of the original steady-state signal (so-called half peak time) was identified for each sample. Trolox was used as a standard inhibitor. The results obtained were expressed as *µ*mol of peroxyl radical trapped by one litre of plasma.

### 2.4. Statistics

Data were examined using the Student's *t*-test, and *P* values below 0.05 and 0.01 were considered statistically significant.

## 3. Results

### 3.1. Arthritis Caused Priming of Neutrophils

**Figure 3:**
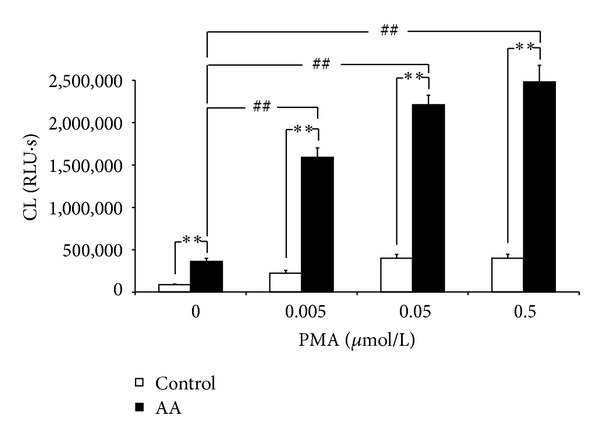
Priming of neutrophils in arthritic rats. Arthritic animals (AA) showed significantly higher spontaneous (0) and PMA-stimulated ROS production (**P < 0.01 AA versus control). PMA caused dose-dependent increase in ROS production which was significantly higher in comparison with spontaneous ROS production in arthritic rats (^##^ *P* < 0.01 AA-stimulated versus AA-spontaneous). Values are mean ± SEM, *n* = 10. RLU∗s: relative light units multiplied by time (seconds).

Arthritic animals (rats with induced arthritis with solvent treatment only) showed significantly (**P < 0.01) higher production of ROS in blood in nonstimulated (spontaneous) and PMA-stimulated conditions in comparison with healthy controls (rats with solvent treatment only) (Figure 3). In arthritic animals the use of PMA increased significantly (^##^ *P* < 0.01) the production of ROS in comparison with spontaneous ROS production. The effect of PMA stimulation was concentration dependent. The highest concentration of PMA used (0.5 *µ*mol/L) did not induce much higher ROS production in comparison with 0.05 *µ*mol/L PMA. In the further analysis, we therefore discuss spontaneous and 0.05 *µ*mol/L PMA-stimulated samples.

### 3.2. Effect of Pterostilbene on Neutrophil ROS Production in Arthritic Rats

**Figure 4:**
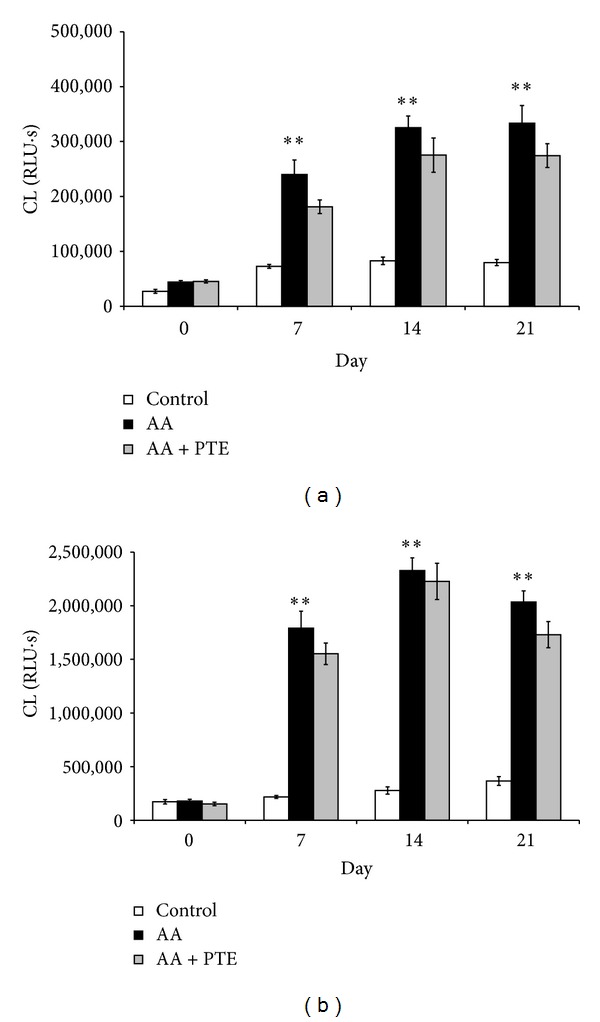
Effect of pterostilbene on ROS production in arthritic rats. (a) Spontaneous and (b) PMA-stimulated (0.05 *µ*mol/L) whole blood chemiluminescence (CL) of healthy (control), arthritic (AA), and pterostilbene treated arthritic rats (AA + PTE). Chemiluminescence was measured at the beginning and every 7 days for 21 days of the experiment. Values are mean ± SEM, *n* = 10, **P < 0.01 AA versus control. RLU∗s: relative light units multiplied by time (seconds).

Nonstimulated (spontaneous) neutrophil ROS production in arthritic rats is evident already on day 7 and was significantly (*P* < 0.01) higher in comparison with healthy controls during the whole experiment, reaching its maximum on day 21 (Figure 4(a)). Although the arthritic rats treated with pterostilbene exhibited lower values of spontaneous ROS production, these changes were not significant compared to the nontreated arthritic group.

The profile of PMA-stimulated neutrophil ROS production was similar to the spontaneous one (Figure 4(b)), with a maximum value on day 14 and with no significant reduction caused by pterostilbene.

### 3.3. Effect of Pterostilbene on Number of Neutrophils and Neutrophil Activity in Arthritic Rats

**Figure 5:**
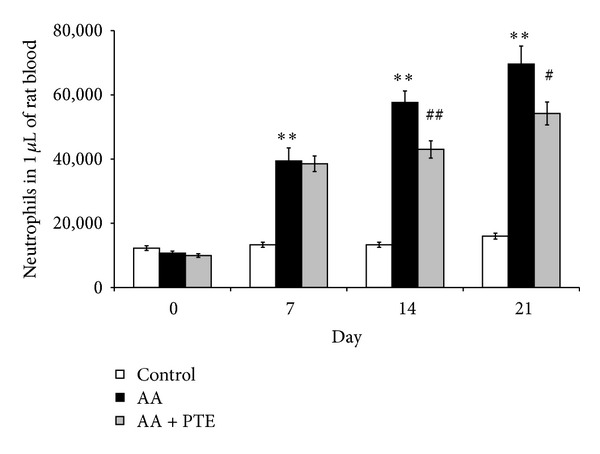
Pterostilbene downregulated the number of neutrophils in arthritic rats. Number of neutrophils in 1 *µ*L of rat blood in healthy (control), arthritic (AA), and pterostilbene treated arthritic rats (AA + PTE) measured at the beginning and every 7 days for 21 days of the experiment. Values are mean ± SEM, *n* = 10, **P < 0.01 AA versus control, ^#^ *P* < 0.05, ^##^ *P* < 0.01 AA + PTE versus AA.

Figure 5 shows the change in the number of neutrophils in healthy, arthritic, and in pterostilbene treated arthritic animals. Arthritic animals demonstrated a significant increase (*P* < 0.01) in neutrophil numbers from day 7 till the end of the experiment, with a maximum value on day 21. A significant decrease was observed within the arthritic group treated with pterostilbene compared to the arthritic group on days 14 and 21 (*P* < 0.01 and *P* < 0.05, resp.).

**Figure 6:**
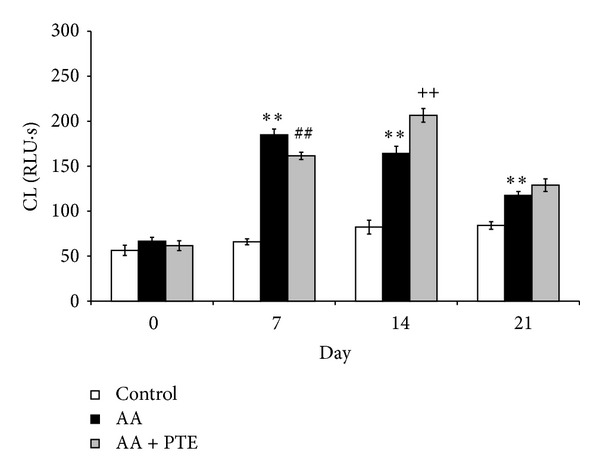
Effect of pterostilbene on neutrophil activity in arthritic rats. PMA (0.05 *µ*mol/L) stimulated ROS production shown as chemiluminescence (CL) per 1 neutrophil in whole blood of healthy (control), arthritic (AA), and pterostilbene treated arthritic rats (AA + PTE) measured at the beginning and every 7 days for 21 days of the experiment. Values are mean ± SEM, *n* = 10, **P < 0.01 AA versus control, ^##^ *P* < 0.01 AA + PTE versus AA for inhibition, ^++^ *P* < 0.01 AA + PTE versus AA for elevation. RLU∗s: relative light units multiplied by time (seconds).

By normalisation of the oxidative burst to the number of neutrophils (neutrophil activity), we found a significant (*P* < 0.01) increase in PMA-stimulated neutrophil activity in arthritic rats compared to healthy controls, with a maximum value on day 7 (Figure 6). Neutrophils from pterostilbene treated arthritic animals showed significantly decreased activity on day 7 compared to arthritic controls. After day 14, however, neutrophils from pterostilbene treated arthritic animals showed higher activity compared to arthritic animals. No significant differences were seen in spontaneous neutrophil activity in healthy, arthritic, or pterostilbene treated arthritic rats (data not shown).

### 3.4. Effect of Pterostilbene Treatment on TRAP Levels in Rat Plasma

**Figure 7:**
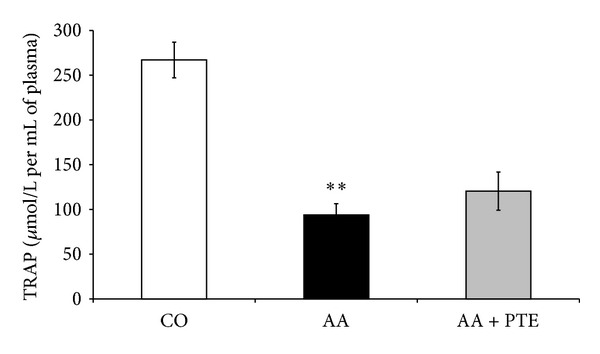
Effect of pterostilbene on TRAP in arthritic rats. Total peroxyl radical trapping capacity (TRAP) in plasma of healthy (CO: control), arthritic (AA), and pterostilbene treated arthritic rats (AA + PTE) measured at the end of the experiment. Values are mean ± SEM, *n* = 10, **P < 0.01 AA versus control. The values are expressed as *µ*mol of peroxyl radical trapped by one litre of rat plasma.

The total peroxyl radical trapping capacity of plasma (TRAP) was determined in the control, arthritic, and pterostilbene treated arthritic groups. The arthritic rats showed a significant (*P* < 0.01) decrease in TRAP (94 *µ*mol/L) compared to the healthy control group (267 *µ*mol/L). Pterostilbene treated arthritic rats showed nonsignificant increase in the total peroxyl radical trapping capacity of plasma (120 *µ*mol/L) compared to the arthritic control group (Figure 7).

## 4. Discussion

The formation of reactive oxygen species (ROS) in neutrophils is one of the essential microbicidal mechanisms in an organism. When produced in high amounts, these highly reactive substances may contribute to tissue injury. This is seen in chronic inflammatory diseases (e.g., rheumatoid arthritis) or ischaemic-reperfusion injury [3, 4, 29]. In this work we examined the potential protective effects of pterostilbene on neutrophil ROS production in adjuvant arthritis—a model of neutrophil-dominant chronic autoinflammatory disease. Pterostilbene was chosen because of its inhibitory effects on neutrophil ROS production *in vitro* [20].

Adjuvant induced inflammation significantly increased the production of ROS and the number of neutrophils in arthritic animals. However, the increased spontaneous (nonstimulated) ROS production in arthritic animals is due to the increase of blood neutrophils rather than the increase in neutrophil activity. This is because proinflammatory cytokines involved in the development of rheumatoid arthritis cause only weak ROS production in neutrophils [30]. Different cytokines present in the synovial fluid induce priming of the neutrophils [3]. By normalisation of ROS production to the number of neutrophils (neutrophil activity), there was no difference between the activities of nonstimulated neutrophils from arthritic and healthy rats. But primed neutrophils have upregulated ROS production when exposed to a secondary stimulus such as PMA [30]. By using PMA, the ROS production in whole blood of arthritic rats was approximately 7 times higher than that in control animals, and the neutrophil activity was significantly increased. Thus, the increase in PMA-stimulated ROS production in arthritic rats is due to an increase in neutrophil number and to an increase in activity of primed neutrophils.

Administration of pterostilbene (30 mg/kg, daily, p.o.) decreased significantly the number of neutrophils in arthritic rats but pterostilbene had only limited effect on ROS production and neutrophil activity. However, the effect of pterostilbene on decreasing the number of neutrophils seems to be more important than the effect on neutrophil oxidative burst. This is because we found that the ROS production in arthritic animals is due to the increase of blood neutrophils rather than the increase of neutrophil activity. The higher neutrophil activity in arthritic rats treated with pterostilbene in comparison with nontreated arthritic rats on day 14 could be explained by the fact that pterostilbene on day 14 significantly decreased the number of neutrophils but the ROS production was nearly the same as in arthritic rats. The lower effect of pterostilbene *in vivo* is seen also with TRAP assay, where pterostilbene did not increase significantly the antioxidative capacity of arthritic rat plasma. These results are in contrast to our findings with pterostilbene effects on neutrophil activity *in vitro* and in cell free assays ([20] and unpublished data), suggesting that the mild decrease in ROS production in arthritic animals receiving pterostilbene treatment may be attributed to pterostilbene downregulation of neutrophilia in arthritic rats.

One of the limits of stilbene derivatives and polyphenols is their low bioavailability. The bioavailability of stilbene derivatives depends on the substitution of the hydroxyl groups. Pterostilbene has a higher bioavailability in comparison with resveratrol [31]. The doses of pterostilbene used in *in vivo* studies with rats range from 10 to 40 mg/kg [24–26]. It is questionable whether increasing the pterostilbene dose would increase its bioavailability and thus the effects *in vivo*.

Another stilbene derivative, pinosylvin (*trans*-3,5-dihydroxy stilbene), was more effective in the inhibition of both spontaneous and PMA-stimulated ROS production in arthritic rat blood and significantly increased TRAP in plasma of arthritic animals [32]. Pinosylvin (30 mg/kg, p.o., daily), though not pterostilbene, decreased the hind paw volume (clinical symptom of adjuvant arthritis) and myeloperoxidase (MPO) activity in hind paw joint homogenates of arthritic rats [23, 33]. MPO may be used to assess the infiltration of neutrophils in the joints [33]. Downregulation of the number of neutrophils in arthritic rats was seen also with pinosylvin [32]. We suggest that stilbene derivatives may interfere with cytokine signalling leading to a decrease of neutrophils in arthritic rats. Resveratrol was found to regulate different cytokines and intracellular messengers involved in inflammation [34, 35]. Paul et al. reported that pterostilbene decreased the levels of the proinflammatory cytokines TNF-*α*, IL-1*β*, and IL-4 [36].

Protein kinase C (PKC) is important in the activation of neutrophil NADPH-oxidase and thus in the production of ROS [37]. Inhibition of PKC could be used as a strategy for regulating various diseases involving PKC [38]. Derivatives of resveratrol are able to attenuate the activity or activation of PKC. Pinosylvin, but not pterostilbene, decreased the activation of PKC in neutrophils *in vitro* [32, 39].

In conclusion, stilbene derivatives are effective in the inhibition of neutrophil ROS production but this is structure dependent. The structure influences also the bioavailability of stilbene derivatives. Despite higher activity against neutrophil ROS production *in vitro*, pterostilbene did not decrease significantly the oxidative burst of neutrophils *in vivo*. On the other hand, pterostilbene decreased the number of neutrophils in arthritic rats. Our results contribute to the knowledge of structure-dependent benefits of stilbene derivatives in the management of chronic inflammatory diseases where neutrophils play a role.
